# Recent insights into catechins-rich Assam tea extract for photoaging and senescent ageing

**DOI:** 10.1038/s41598-024-52781-2

**Published:** 2024-01-26

**Authors:** Mayuree Kanlayavattanakul, Mattaka Khongkow, Wannita Klinngam, Puxvadee Chaikul, Nattaya Lourith, Piyaporn Chueamchaitrakun

**Affiliations:** 1 School of Cosmetic Science, Mae Fah Luang University, Chiang Rai, 57100 Thailand; 2 Phytocosmetics and Cosmeceuticals Research Group, Mae Fah Luang University, Chiang Rai, Thailand; 3 National Nanotechnology Center (NANOTEC), National Science and Technology Development Agency, Pathum Thani, Thailand; 4 School of Agro-Industry, Mae Fah Luang University, Chiang Rai, Thailand; 5 Tea and Coffee Institute, Mae Fah Luang University, Chiang Rai, Thailand

**Keywords:** Biotechnology, Chemical biology, Developmental biology

## Abstract

Tea (*Camellia* spp.) is an important medicinal herb. *C. sinensis* var. *sinensis* is the most studied tea variety due to its more preferred flavor than *C. sinensis* var. *assamica* (Assam tea), the less economic importance with more bitter variety. A bitter taste highlights its potential as a candidate source for tea catechins, the health beneficial actives applicable for ageing treatment. Nonetheless, indicative data for tea on UV-induced and senescent ageing remain unclarified. Assam tea extract (ATE) was prepared and standardized in terms of TPC, TFC and TTC. EGCG was HPLC quantified as the prime ATE catechin. In vitro antioxidant activity of ATE was exhibited with ABTS, DPPH and FRAP assays. ATE’s cellular antioxidant activity was indicated in HDFs at a stronger degree than ascorbic acid. The photoaging protection of ATE was evidenced in a coculture of HaCaT cells and HDFs. ATE markedly suppressed UV-induced IL-6, IL-8, MMP-1 and MMP-9 expressions. The proficiency of ATE targeting on senescent ageing was demonstrated in an ex vivo human skin model, where IL-6 and MMP-1 expressions were suppressed, whilst hyaluronic acid and collagen syntheses were promoted. ATE was chemically stabled as indicated by the catechin contents and color parameters following 6 months storage under conditions recommended for topical product. ATE enriched in catechins warrants its applicability as a new generation of photoaging protectant agent promising for the prevention and treatment for senescent ageing. The findings indicate the proficiency of ATE for innovative anti-ageing agent.

## Introduction

Tea (*Camellia* spp.) is an important medicinal herb. It is also a popular beverage that has long been regarded as a natural source of beneficial health and therapeutic actives. The bud with two tender leaves of *C. sinensis* (*C. sinensis* var. *sinensis* and *C. sinensis* var. *assamica* or Assam tea), are commonly picked and further processed into green, black and oolong teas to flavor the beverages with different tea polyphenols and provide a variety of health benefits^1^. The health benefits of tea are widely recognized and manifested by tea polyphenols, particularly catechins^2,3^, the antioxidants with UV protection potential^4^. Surprisingly, indicative evidence on tea and catechins for skin ageing, which multiplied by oxidants and UV-induced effects, is limited.

Oxidative stress crucially initiates and severely propagates adverse events including ageing, against good health conditions^5^. UV exposure induces oxidative stress escalating ageing^6,7^. Oxidants intensify the activities of proteolytic enzymes, resulting in degradation of the extracellular matrix (ECM), i.e., collagen and elastin fibers that are closely associated with the elasticity and tensile strength of the skin. The ECM is degraded by matrix metalloproteinases (MMPs). The functions of these enzymes (MMP-1 to MMP-28) are accelerated with age, radicals, and inflammatory mediators. Accordingly, the deactivation, inhibition or suppression of MMPs and stimulation of ECM synthesis enzymes are regarded as the leading strategy in the management of ageing with antioxidant application is regarded as a major treatment^8^. In addition to the gold standard of treating ageing with antioxidants, prevention or protection against photoaging is recently accounted as the key approach combating ageing^6^ in regards with the current environmental circumstances^9^. Nonetheless, the ideal photoaging prevention/protection is abided with sunscreen or sun production products of which, the potential agent for photoaging treatment is of significance for the new generation of anti-ageing agents^5^.

Tea extract has been incorporated into a variety of cosmetics^3,10^ with the claimed antioxidant and UV protection activities^4^ that ruled by its major components, i.e., catechins. Different catechin derivatives have different biological activities, among which epigallocatechin-3-gallate (EGCG) is regarded as a prominent catechin with high health-promoting effects^9^. Nonetheless, a precise mechanism of tea extract and its EGCG against photoaging, which exacerbates oxidative stress and accelerates chronologic, extrinsic and senescence ageing, is barely revealed. Numerous studies are exclusively undertaken in *C. sinensis* var. *sinensis* in accordance with its more preferred flavor that makes it more expensive than Assam tea. Application of Assam tea in a certain high value product, i.e., cosmetic industry, is considerable underestimate due to its gap of supportive and indicative study in contrary to *C. sinensis var. sinensis*.

In this context, Assam tea extract (ATE) with a high catechin content was objectively prepared and standardized in terms of the active contents. The biological activities associated with photoaging protection were investigated in vitro, in human dermal fibroblasts (HDFs), cocultures of HaCaT cells and HDFs, and in a human ex vivo skin model in an order to concrete safety and efficacy of ATE for anti-ageing utilization. In addition, the chemical stability of the extract was examined under different storage conditions recommended for topical product formulations, and the physicochemical stability was monitored in parallel. ATE presented here is therefore warranted for innovative anti-ageing products targeting for photoaging and premature or senescent ageing treatments with the full spectrum of relevant chemical and biological profiles. Moreover, Assam tea is highlighted as a promising source for the specialty ingredients, catechins, especially EGCG that are applicable for varieties of health promotion products.

## Results

### Assam tea extraction and quantification of actives

**Figure 1:**
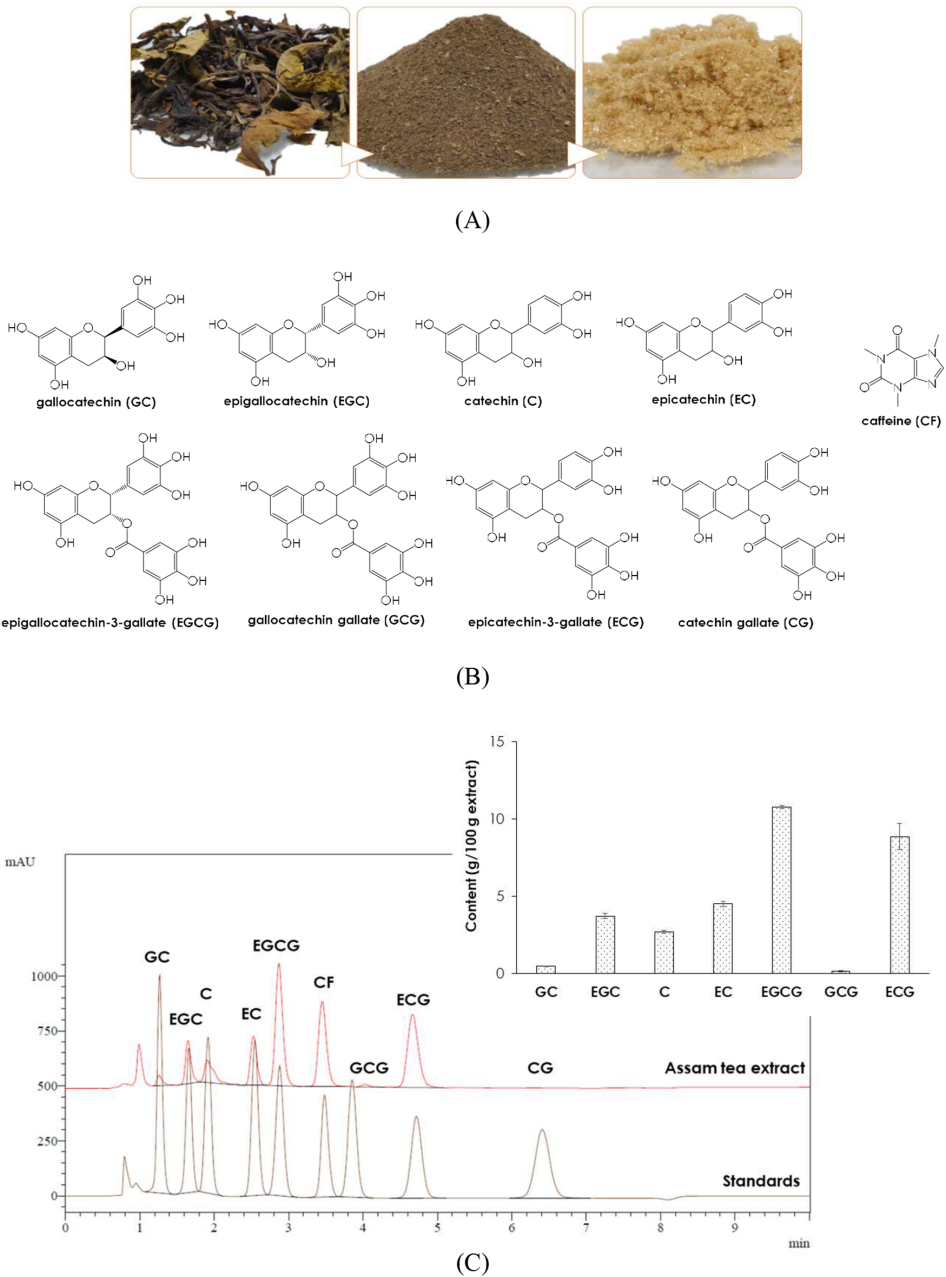
Assam tea leaves, powder and extract (**A**), the studying catechins and caffeine (**B**) and catechins profiles of the extract (**C**).

Assam tea was collected and its powder was extracted giving a light brown color extract as shown in Fig. 1A, in which, the color-indicating parameters of the extract are shown in Table 1. The active components of ATE were quantified in terms of its total phenolics (TPC), total flavonoids (TFC), and total tannins (TTC) contents (Table 1).

**Table 1:** Extractive yield, color and active principles content of Assam tea extract.

Parameter		Value
Extractive yield (%)		38.94 ± 0.11
Color	L*	32.50 ± 0.01
	a*	0.49 ± 0.01
	b*	5.26 ± 0.04
TPC (mg gallic acid/ g)		802.44 ± 4.89
TFC (mg quercetin/ g)		195.02 ± 13.14
TTC (mg tannic acid/g)		317.09 ± 1.78

Quantification of the extract’s catechins (Fig. 1B) was performed by a standardized HPLC method^11^, EGCG was noted as the predominant (p < 0.001) catechin in the extract, while GCG was the least predominant (Fig. 1C). In addition, caffeine was detected at a high level (10.90 ± 0.36 g/100 g extract).

### In vitro antioxidant activities

The in vitro activities of ATE were examined using 2,2′-azino-bis(3-ethylbenzothaiazoline)-6-sulfonic acid) (ABTS), 1,1-diphenyl-2-picrylhydrazyl (DPPH) and ferric reducing ability of plasma (FRAP) assays. ATE was noted for its potent radical scavenging activity indicated with these 3 different assays. ABTS and DPPH radicals were terminated by ATE at IC_50_ of 4.60 ± 0.18 and 7.13 ± 0.50 μg/mL, although ATE’s activity was less potent than that of the standard ascorbic acid (3.81 ± 0.11 and 3.40 ± 0.06 μg/mL, respectively) as summarized in Table 2.

**Table 2:** In vitro antioxidant activities of Assam tea extract.

Assays	Assam tea extract	Ascorbic acid
ABTS (IC_50_, µg/mL)	4.60 ± 0.18	3.81 ± 0.11
DPPH (IC_50_, µg/mL)	7.13 ± 0.50	3.40 ± 0.06
FRAP (EqC, g FeSO_4_/g)	33.20 ± 0.73	–

### Safety and cellular antioxidant activity in human dermal fibroblasts (HDFs)

**Figure 2:**
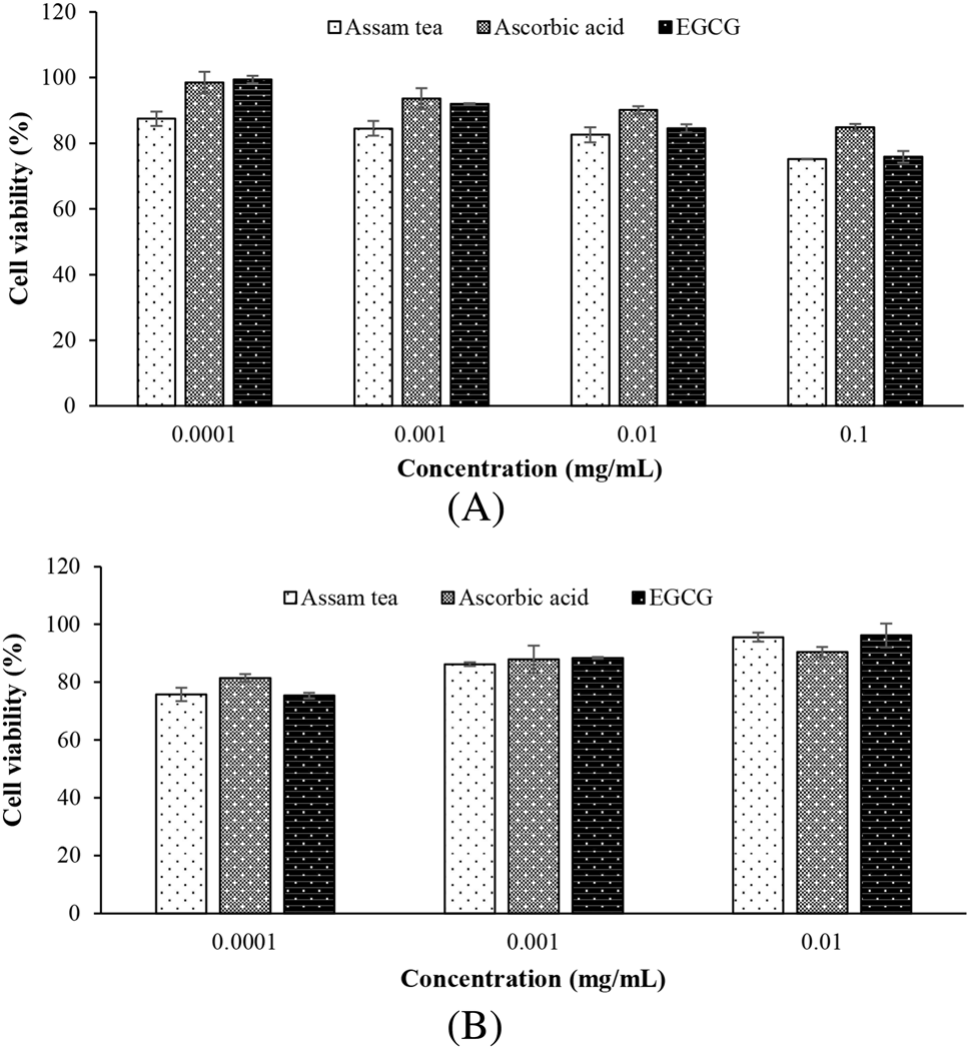
Safety (**A**), cellular anti-oxidant activity (**B**) of the extract examined in HDF.

The extract was first screened for its toxicity to HDFs (Fig. 2A) compared with the standard ascorbic acid and its major catechin, EGCG. At concentrations of 0.1–10 µg/mL, the extract was indicated to be negatively cytotoxic to the cell, producing cell viabilities greater than 80% confers noncytotoxic to the cell^12^, which is comparable with the standards. This range was accounted as the safe concentrations, and further challenged on antioxidant activity. Cellular antioxidant activity of the extract was evidenced at the noncytotoxic concentrations (Fig. 2B), where the antioxidant action of the extract and EGCG by means of protection against oxidative stress was comparable. It should be noted that the extract and its marker, EGCG, were significantly more potent (p < 0.05) than the standard ascorbic acid at the highest test concentration (10 µg/mL).

### Safety and anti-ageing activity in coculture of HaCaT and HDF cell lines

**Figure 3:**
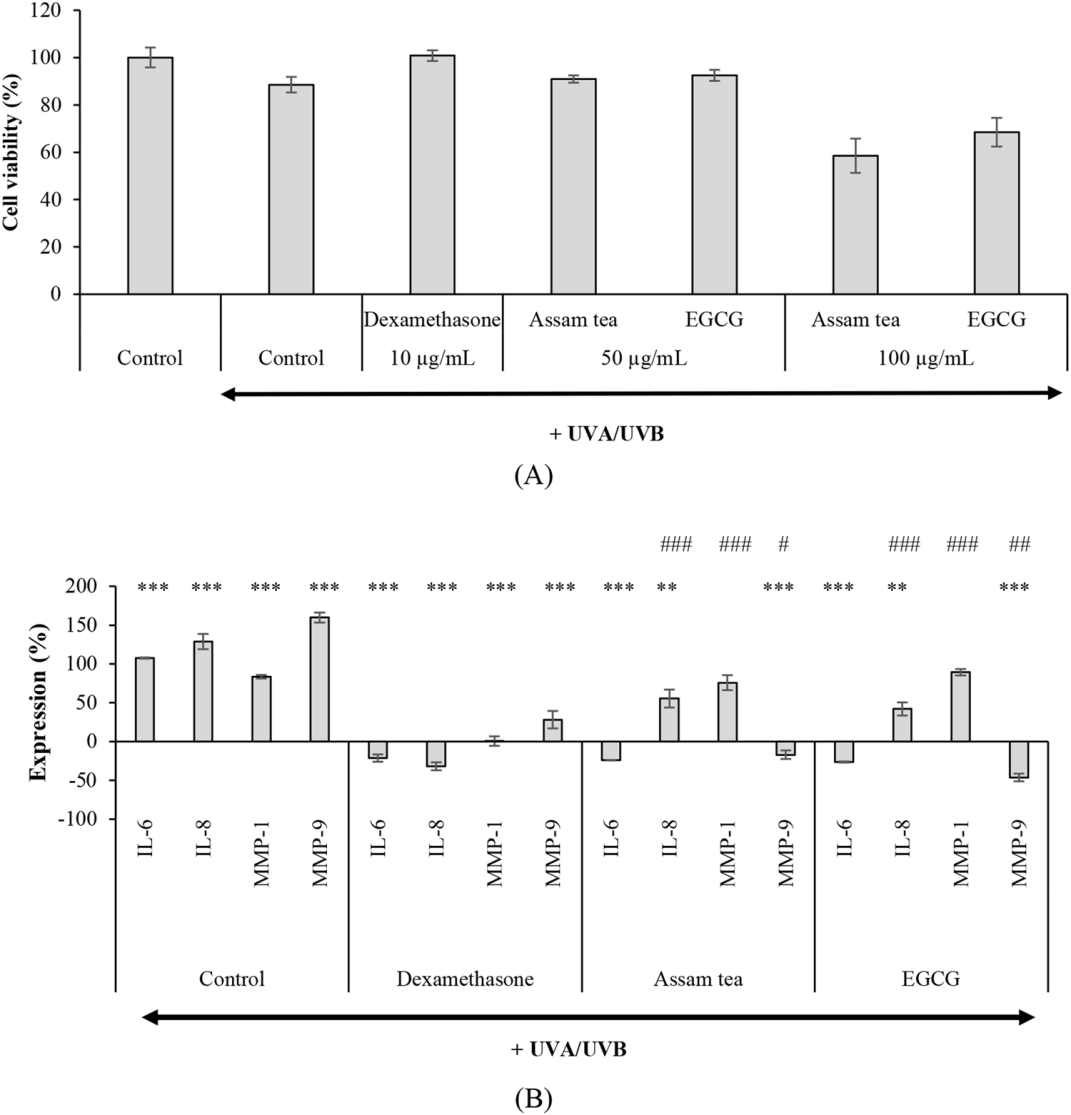
Safety (**A**) and photoaging protection activities of the extract (**B**) examined in a coculture system (*p < 0.05, **p < 0.01, ***p < 0.001 compared with untreated samples with UV-exposure group; ^#^p < 0.05, ^##^p < 0.01, ^###^p < 0.001 compared with dexamethasone).

The coculture model was UV-induced cytotoxic. ATE (50 µg/mL) and EGCG were shown to protect cocultured HaCaT cells and HDFs against UV damage, while a twofold increases in the dose caused cytotoxic (Fig. 3A). The benchmark dexamethasone (10 µg/mL) has been noted to protect the skin cocultures from photoaging, resulting in cell viability similar to that in the control group without UV exposure.

Activities of the extract against inflammatory mediators and MMPs were therefore assessed at noncytotoxic concentrations (Fig. 3B). ATE significantly (p < 0.01) suppressed the expression of IL-6, IL-8, MMP-1 and MMP-9 following UV exposures, although less potent than the benchmark dexamethasone (p < 0.05).

### Safety and anti-ageing activity in human ex vivo skin model

**Figure 4:**
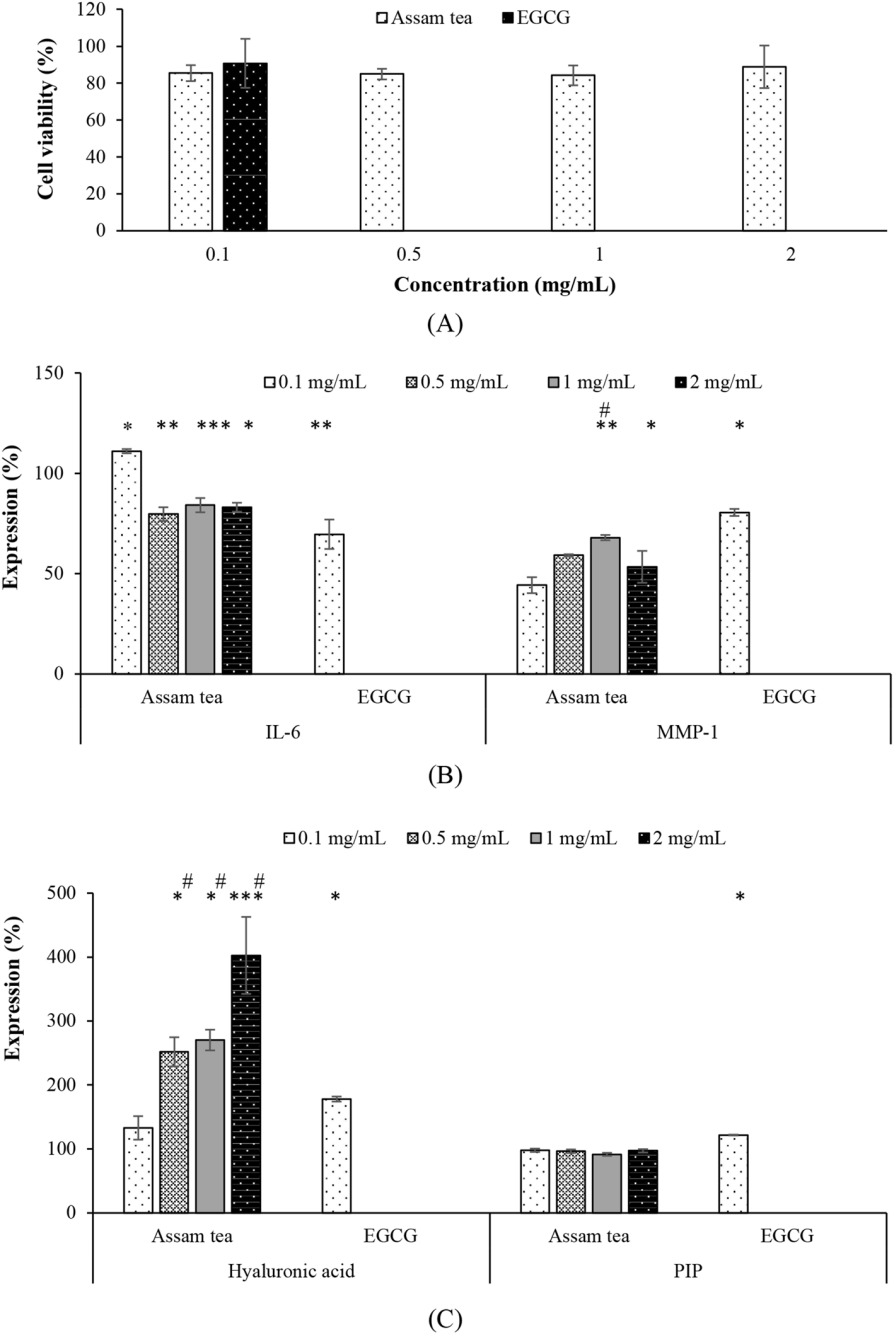
Safety (**A**), IL-6 and MMP-1 (**B**), and hyaluronic acid and PIP (**C**) expressions examined in human ex vivo skin model (*p < 0.05, **p < 0.01, ***p < 0.001 compared with control untreated group; ^#^p < 0.05 compared with EGCG).

Preliminarily, a safe dose of ATE was examined, and was shown to be noncytotoxic at 0.1–2 mg/mL, while the safety concentration of EGCG was indicated at 0.1 mg/mL (Fig. 4A). Therefore, the biological activity in a human ex vivo skin model in terms of IL-6 and MMP-1 inhibitions and hyaluronic acid and PIP stimulations was examined at the safe concentrations.

ATE and EGCG were proved upon their anti-ageing activity suppressing IL-6 and MMP-1 expressions in senescent cells (Fig. 4B) significantly (p < 0.05). Furthermore, ATE and its active compound, EGCG, capable (p < 0.05) of promoting cellular hyaluronic acid production and PIP expression (Fig. 4C).

### Stability of Assam tea extract

**Figure 5:**
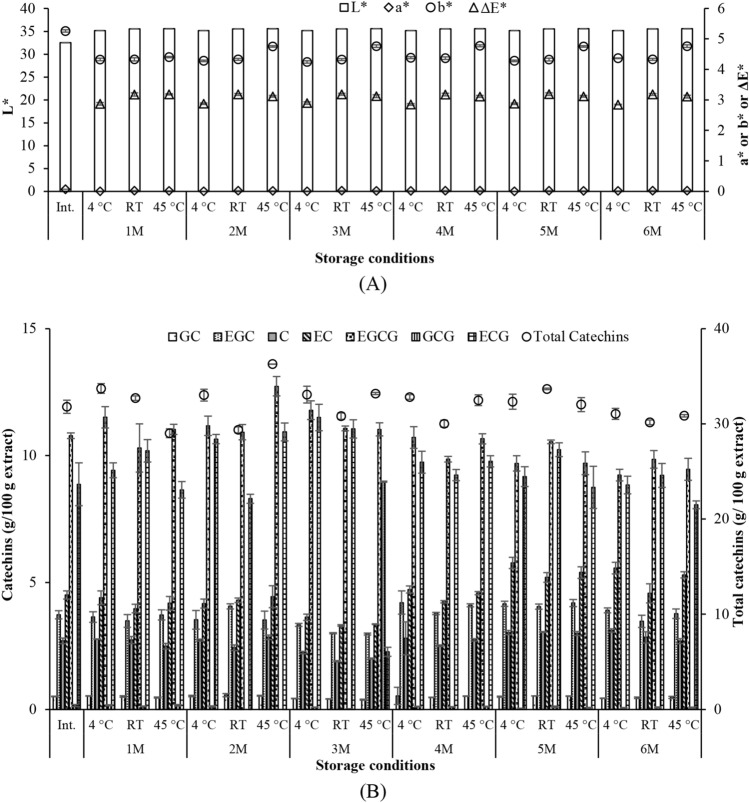
Stability of Assam tea extract following 6 months storage under different conditions.

ATE was stored under different recommended conditions for topical products including cosmetics^13,14^. Color parameters indicating the physicochemical properties of the extract were unchanged (Fig. 5A). In addition, catechins were quantified with the standardized HPLC^11^ as above confirming chemical stability of the extract (Fig. 5B). Which, the color stability was conformed with the catechin content data.

## Discussion

Tea (Fig. 1A) is a well-known medicinal herb that is popularly processed into beverages and several household and personal care products. *C. sinensis* var. *sinensis* is the most studied tea variety due to its preferred flavor, but it is more expensive than Assam tea, the bitter variety. Assam tea is more bitter than *C. sinensis* var. *sinensis* due to its higher tannin content^15^. The bitterness and astringency of tea are governed by catechins^16^. Thus, Assam tea is a potential candidate source of health-beneficial catechins applicable for innovative health promotion products, including anti-ageing formulations targeting on photoaging and senescent ageing. Which, this sort of anti-ageing agent is continuously growing in demand^5^ in the cosmetic market especially those of natural derived ones^17^.

Catechins are the key bioactive components of tea. EGCG has been highlighted as the major catechin among tea catechin derivatives (Fig. 1B) due to its versatile applications^1,10^. Caffeine is a biologically active compound found in tea, and finds its applicability to different fast-moving consumer goods (FMCGs), i.e., nutraceuticals, cosmetics and personal care products. Caffeine can stimulate microcirculation and accelerate lipolysis, giving its application prospects as an anticellulite product^18,19^. Thus, an extraction procedure was prominently designed to generate ATE with a maximized catechins content, and caffeine was additionally determined in the extract as well.

In an order to achieve a sustainable utilization for the less economic importance Assam tea with the least interruption on food safety, the tea leaves that were not supplied for beverage productions were harvested, prepared and extracted. Catechins of Assam tea were extracted by a modified method using aqueous EtOH. Which, 50% EtOH has been reported to be the most optimized solvent^20,21^ for catechins extraction, and 90 min was the efficient time^21^. The extracting condition yielded the extract with a specification set forth to the topical industrial application as depicted in Table 1. The major polyphenols in tea are flavonoids, tannins and catechins, and their contents were fundamentally quantitated by determining the TPC. The extraction presented in this study afforded ATE with a higher TPC than the previous reports on green tea extracted by ultrahigh pressure, microwave, ultrasonic, Soxhlet and reflux (390.68–576.02 mg gallic acid/g) using the same solvent, plant/sample ratio^21^ and extraction time (18.70–68.60 mg gallic acid/g)^22^. In addition, the modified extraction procedure produced the extract in a greater TPC than an extraction with a higher plant per solvent ratio (1:2 w/v), a greater shaking speed (240 rpm) and a longer time of extraction (24 h) using 80% MeOH (109.85–216.16 mg gallic acid/g)^23^ or the extract that was prepared by 80% EtOH maceration for 2 h (21.3–31.6 mg gallic acid/g)^24^. Furthermore, agricultural practices relating with cultivation as well as geographical conditions may vary the phytochemical constituents of the tea. Thus, the presented method offers an efficient preparation method for tea extract enriched with catechins by a concise method and having feasibly for an industrial practice. In addition, Assam tea cultivated in Chiang Rai, Thailand is highlighted as the promising source of natural polyphenols as exhibited with its TPC.

The extract was additionally TFC and TTC examined. These values were accountable for routine quality control of the extract in addition to TPC that was fundamentally quantified. Tea polyphenols, especially tannins and catechins play a significant role in the bitterness and astringency, which are monitorable by TTC and TFC. ATE of this study was noted on its greater TFC than the previous report (14.3–38.18 mg quercetin/g)^23^. The TTC is an important constituent in anti-inflammatory herbal extracts^25^. In addition, tannins are astringent, and essential for reducing skin pores and greasiness, and applicable for use in skin care cosmetics, i.e., those that treat acne^26^, and are used for skin lightening^27^ and sun care products^28^. The presenting study afforded ATE that higher in TTC than the previous report (6.0–7.45 mg tannic acid/g)^24^. ATE was noted to have a greater TTC than green apple rind (30.48 ± 0.38 mg tannic acid/g), which has anti-DPPH activity^29^, and a higher TTC than Ceylon spinach (4.18 ± 0.09 µg tannic acid/ g), which was clinically shown to be a promising skin hydrating agent^30^. Accordingly, ATE would be applicable for acne care and hydrating products development.

The prime active component of ATE was confirmedly EGCG (Fig. 1C), and caffeine was also detected at a high level under the presented extracting conditions. Accordingly, the in vitro antioxidant activities of ATE related to its active constituents were screened using ABTS, DPPH and FRAP assays (Table 2). Interestingly, the anti-DPPH activity of ATE was more potent than that of the Brazilian Assam tea extracts obtained by ultrasound-assisted extraction with IC_50_ values between 8.33 and 16.10 µg/mL^31^. In addition, it was indicated to be stronger in DPPH quenching ability than the Iranian and Ethiopian Assam tea (IC_50_ = 167.11–505.5 and 7.3–64.0 µg/mL) that were constituted with lower TPC, TFC and TTC contents^23,24^. In comparison with the green apple rind extract, which had a lower TTC than ATE, the DPPH scavenging activity was also less potent (IC_50_ = 668.89 ± 12.40 mg/mL)^29^.

Scavenging of DPPH mechanisms by a hydrogen atom transfer, which finds more sensible with those of smaller molecular weight phenolics in a comparison with ABTS. Potent ABTS^•+^ scavengers, greater molecular weight phenolics with a less substitution of the hydroxyl groups, donate one anion radical or two neutral radicals or electrons to compensate the cation radical of ABTS^•+^, while the oxidized intermediates in the peroxidation are monitorable by an ability to reduce Fe^3+^ to Fe^2+^, i.e., FRAP^32^. Which is theoretically consistent with the constituents of ATE, catechins. In addition, the studying ATE was more potent in reducing power than the Iranian extracts (0.2–2.1 μg/g)^24^. Accordingly, antioxidant activity of ATE is indicatable by the active principles, i.e., TPC, TFC and TTC, and applicable for its quality control practice.

Reactive oxygen species are accepted as the agents responsible for severe cellular damage and have been implicated in the causation of several degenerative diseases, including cutaneous ageing. Accordingly, antioxidants are accepted as major therapeutic ingredients for skin ageing, among which herbal-derived agents are significant^8,17^ amidst the current awareness of sustainable product development^33^. In accordance with the in vitro antioxidative ATE, we proposed that ATE would mitigate a cellular oxidative stress. Thereafter, antioxidant activity of ATE in a cellular model relevant to skin, i.e., HDFs, was undertaken. The protective effect of ATE against cellular oxidative damage was evidenced by a cell viability of more than 80% after treatment. Furthermore, the cellular antioxidant activity of ATE increased in a dose-dependent manner (Fig. 2B) and was significantly better than the benchmark ascorbic acid at the maximum tested concentration (p < 0.01). It should be noted that the antioxidant performance of ATE was comparable (p > 0.05) to that of its active catechin component, EGCG. Thus, ATE is warranted on its ability to mitigate the deleterious effects caused by cellular oxidative stress.

Th extract, which has shown great promise as an anti-ageing agent in in vitro and HDFs assessments, was next questioned whether it could protect dermal cells from UV-induced photoaging and how it works. Extrinsic skin ageing primality arises after UV exposure, and almost 80% of facial skin ageing is attributed to such exposure. Accordingly, photoaging protectant agent is emerging as a significant new generation active promising for anti-ageing product^5^.

UV-induced oxidative stress upregulates MMPs, especially MMP-1 (collagenase) and MMP-9 (gelatinase)^9,34^, the enzymes that degrade collagen, gelatin, elastin, and fibronectin^35^. Dermal damage is induced by shorter wavelengths UV exposure (UVB), which are absorbed by the epidermis prior to keratinocytes receiving irradiation. On the other hand, longer wavelengths (UVA) penetrate the skin and interact with epidermal and dermal cells^8^. In addition, UV exposure induces coregulated factors after MMPs are expressed, include cytokines, i.e., IL-6 and IL-8^36^, which have several inflammatory effects, and are directly involved in the ageing of skin^37^. Furthermore, these cellular oxidative events exacerbate the nonproteolytic and common proteolytic activities. Which, MMP-1 and MMP-9 are predominately activated by UV exposure in keratinocytes and fibroblasts. Moreover, MMPs are involved in numerous skin diseases, including inflammatory processes, which exacerbate skin ageing^38^. Nonetheless, the studied models were chiefly in either keratinocytes or fibroblasts^17^. ATE’s activity was thereafter challenged in a coculture model for the first time.

In regards with protective effect of ATE against cellular oxidative damage aforementioned in HDFs, its ability in protecting dermal cells from photodamage was determined in the coculture model. ATE (50 µg/mL) and EGCG were shown to protect cocultured HaCaT cells and HDFs against UV damage, while a twofold increases in the dose caused cytotoxicity evidenced with cell viabilities of less than 80% as shown in Fig. 3A. UV exposure clearly (p < 0.001) upregulated the expression of IL-6, IL-8, MMP-1 and MMP-9 (Fig. 3B). ATE markedly suppressed UV-induced IL-6, IL-8, MMP-1 and MMP-9 expressions. Inhibitory effects of ATE against IL-6, IL-8 and MMP-1 were comparable to that of EGCG (p > 0.05). However, it’s anti-MMP-9 activity was weaker (p < 0.05) than EGCG. In a comparison with the benchmark dexamethasone, the proficiency of ATE and EGCG targeting on IL-6 was comparable (p > 0.05), but weaker against IL-8, MMP-1 and MMP-9 (p < 0.05). Interestingly, the presented ATE reduced IL-6 and IL-8 expressions by 2.7- and 1.5-fold, respectively, which was stronger than that in a previous study in keratinocytes exposed to PM10 and treated with 10 µM EGCG (1- and less than onefold reductions)^39^. A report in fibroblasts also showed that 50 µM EGCG suppressed MMP-1 and MMP-9 expressions^40^. In addition, 1 µM EGCG was reported to inhibit heat shock-induced MMP-1 expression[41]. Thus, ATE that has been indicated to protect skin from UV irradiation would maintain skin homeostasis against adverse airborne particulate matter exposure and heat as well.

Dermal fibroblast senescence is caused and accumulated by skin ageing. Alterations and degradation of the ECM are associated with ageing^42^. ATE was indicated to be effective against ageing, particularly photoaging as declared above. We proposed that ATE could ameliorate senescent ageing. Activity of ATE against senescent ageing was further challenged using an ex vivo skin model. A human ex vivo skin or tissue biopsy culture model is a valuable tool to prove the potency of a candidate active and allows the replacement of an animal model^43^.

Preliminarily, safe doses of ATE on a human ex vivo skin model were examined, and were shown to be noncytotoxic at 0.1–2 mg/mL, while that of EGCG was at 0.1 mg/mL (Fig. 4A). Therefore, the biological activity in this cellular model in terms of IL-6 and MMP-1 inhibitions, and hyaluronic acid and PIP stimulations was examined at the safe concentrations. IL-6 and MMP-1 are markers of inflammation and collagen breakdown. Accordingly, ATE’s activity against these markers were challenged together with its capability on cellular promotions of the ECM components, i.e., hyaluronic acid and PIP. ATE and EGCG significantly suppressed IL-6 and MMP-1 expressions in the senescent cells, except for the extract at 0.1 mg/mL (Fig. 4B). The inhibitory effect of ATE (0.5, 1 and 2 mg/mL) against IL-6 was comparable (p > 0.05) to that of EGCG. Interestingly, the anti-MMP-1 activity of ATE was significantly (p < 0.05) greater than that of EGCG. In addition, ATE was clearly (p < 0.05) better performed than EGCG in promoting hyaluronic acid production, but less potent than EGCG in promoting PIP expression (Fig. 4C).

Taken into account, ATE was indicated to maintain skin homeostasis following oxidative stress and UV exposure diminishing photoaging effects and senescent ageing. The prospects of using ATE in an innovative anti-ageing product were warranted with its chemical and biological profiles presented above. To solidify its applicability to topical product formulations, the stability of the extract is of crucially important^12^. ATE was challenged under different recommended conditions applicable for topical products^13,14^. Color parameters indicating the physicochemical properties of the extract were unchanged (Fig. 5A) when ΔE was lower than the visually detectable range^33,44^. The color stability was conformed with the catechin content data (Fig. 5B). However, it should be noted that following 6 months of storage, the catechin contents decreased (2–5%). Nonetheless, the relative changes from the initial were less than 20% that falls in the stable range for cosmetics^33^. To enhance the stability of ATE with a minimized of preservative used, an appropriate system/formulation should be developed to maintain the stability of its pharmacologically active molecules.

In summary, ATE enriched in catechins, especially EGCG, was prepared and standardized. ATE was verified on its anti-ageing property targeting for photoaging and senescent ageing in coculture and ex vivo skin models for the first time. Innovative anti-ageing product profiled with the new generation of photoaging protectant agent derived from Assam tea presented is encouraged to be developed, and assessed upon the product stability, safety and anti-ageing proficiency and preference in human volunteers. Furthermore, Assam tea is a warranted source for the specialty ingredients, catechins, that are applicable for varieties of health promotion products. In addition, the routine quality control of the extract would be practically performed abided with the chemical profile of ATE presented.

## Materials and methods

### Materials

All chemicals used were analytical grade unless otherwise specified. The solvent for extraction, EtOH was purchased from Merck (Darmstadt, Germany). ABTS, DPPH FRAP reagents for antioxidant activity assessments were supplied by Fluka (MO, USA), as were the ascorbic acid and FeSO_4_ standards including those that were used for TPC, TFC and TTC analysis. Reference compounds for HPLC analysis were purchased from Fluka (≥ 99% HPLC grade). The HPLC standards were diluted at various concentrations in AcCN (Labscan, Gliwice, Ireland). De-ionized water was prepared using a Milli-Q water purification system (Millipore, MA, USA). Media and supplements for cellular assessments were from Gibco (NY, USA). SRB was from USB (OH, USA), and DMSO (Sigma, MO, USA) was used to solubilize the cells.

### Assam tea extraction

The leaves of *C. sinensis* var. *assamica* cultivated in Wawee Nature Farm, Wawee Nature Group Co., Ltd. Chiang Rai, was harvested during March 2023. Plant access and collection practices were complied with national guidelines and legislation, i.e., Plant variety protection Act (1999) of Thailand, with the right permits and following good academic practice. Permission for the collection of plant sample was taken from the farm owner; Mr. Wachara Phosuwun. Asst. Prof. Dr. Chaisak Chansriniyom from Faculty of Pharmaceutical Sciences, Chulalongkorn University had identified the plant material with a consent to harvest and collect the plant sample with the voucher specimen of MKCSA 0323. The aforementioned voucher specimen was deposited for further reference at Phytocosmetics and Cosmeceuticals Research Group, Mae Fah Luang University, where access is public and available. The tender 5 leaves and a bud were cleansed with tap water and air-dried under the shade. The dried leaves were pulverized to obtain the powder (Fig. 1A) with the size of less than 0.45 mm. The extraction was done by macerating the plant sample in 50% EtOH (1:20 w/v) under ambient conditions^21^ with shaking for 90 min. The extractant was filtered, concentrated under *vacuo* and further lyophilized to dryness. The extraction was repeated for 2 more times, and the crude extract yield was calculated. In addition, color of the extract was examined (Hunterlab UltraScan VIS) in triplicate on the basis of CIELAB system^33^.

### Total phenolics content (TPC)

TPC was determined using Folin-Ciocalteu assay. In short, the reagent was mixed with Na_2_CO_3_ and absorbance measured using a microplate reader (ASYS, UVM340, UK). The TPC was compared with the gallic acid and expressed as mg gallic acid/ g of the extract. The procedure was repeated in triplicate^45^.

### Total flavonoids content (TFC)

TFC was assessed with aluminum chloride colorimetric assay. Briefly, the sample was mixed with H_2_O and 15% NaNO_2_, incubated, added with 10% AlCl_3_ and 4% NaOH prior to absorbance reading at 570 nm using the microplate reader. The determination was repeated in triplicate. The TFC was calculated by comparing the sample with the standard curve (r > 0.999) obtained from quercetin at different concentrations and expressed as mg quercetin/ g of extract^32^.

### Total tannins content (TTC)

TTC of ATE in DMSO and water was determined by an incubation with 5% Na_2_CO_3_ and 1 N Folin-Ciocalteu for 60 min prior to an absorbance recorded at 725 nm with the microplate reader. Of which, tannic acid was used as the standard^30^.

### HPLC analysis

Catechins, i.e., EC, C, ECG, CG, EGC, GC, EGCG, and GCG were quantified in ATE using HPLC system (Waters Alliance e2695) equipped with PDA detector and a C_18_ 3 μm column (53 × 7 mm) (Platinum C18-EPS). The standards at various concentrations in AcCN were used to prepare calibration curves (r > 0.9995). The samples were successively separated by an isocratic mobile phase consisting of 0.05% aq. TFA (87%) and AcCN (13%) flowed at 2 mL/min. Each catechin was calculated and expressed as g/100 g extract^11^. Quantification of catechins in the extract was performed in three cycles^46^.

### ABTS radical scavenging activity

In vitro activity of ATE to scavenge ABTS^·+^ was determined. Shortly, a mixture of the standard ascorbic acid of different concentrations mixed with the ABTS solution containing 2.450 mM potassium persulfate and absorbance was measured at 750 nm following an incubation for 5 min to generate a calibration curve (r > 0.999). The concentration resulting in 50% inhibition (IC_50_) against the radicals was calculated. The ability of the sample to scavenge ABTS^·+^ was calculated in a comparison with the standard ascorbic acid and expressed as IC_50_. The measurements were done in triplicate^32^.

### DPPH radical scavenging activity

In vitro antioxidant activity was assessed in parallel by means of the DPPH assay. In short, the test sample was mixed with DPPH and reacted for 30 min prior a reduction of DPPH^·^ monitoring at 517 nm. IC_50_ of DPPH^·^ was compared with the standard. All measurements were done in triplicate^32^.

### FRAP assay

The reducing power of ATE was examined. In brief, FRAP reagent was prepared in a 2,4,6-tri(2-pyridyl)-*S*-triazine (TPTZ) solution (10 mM) containing 40 mM HCl, FeCl_3_ (20 mM) and acetate buffer, pH 3.6 (0.3 M). The sample reacted with the FRAP reagent was recorded as absorbance at 595 nm. FeSO_4_ at different concentrations was used for the calibration curve generation. The reducing power was expressed as an equivalent concentration (EqC) to that of 1 mM FeSO_4_. The reducing power was determined in triplicate^32^.

### Safety assessment in HDF

Human dermal fibroblasts or HDF were cultured in DMEM medium supplemented with 10% FBS and 1% penicillin/streptomycin at 37 °C under 5% CO_2_. Cells were grown and harvested by 0.25% w/v trypsin and 0.06 mM EDTA in phosphate buffer saline. Cell cytotoxicity determination was by sulforhodamine B (SRB) assay. Cells were seeded in 96-well plate and incubated for 24 h, treated with different concentrations of the samples for 72 h, fixed, washed and dyed prior to the absorbance measurement at 540 nm with the microplate reader. The cell viability was compared with the control treated with absolute EtOH^47,48^.

### Antioxidant activity in HDF

HDF were incubated for 48 h, treated with the samples and the solvents (absolute ethanol or culture medium) for 24 h prior to treat with the fresh medium containing 150 μM H_2_O_2_ and further incubated for 3 h. The cells were fixed, washed, dyed, and solubilized with 10 mM Tris base and recorded the absorbance at 540 nm for cell viability calculation^47^.

### Safety assessment in a coculture of HaCaT and HDF cell lines

Cellular safety of the samples was firstly examined in a coculture model. In short, HDF were seeded in 48-well plate for 3 days prior to additional seed of HaCaT cells seeded onto the HDF and further incubated for 24 h. Thereafter, the cocultures were treated with the samples, incubated for 24 h, exposed with UVA (1 J/cm^2^) and UVB (30 mJ/cm^2^), incubated for 24 h. Cell viability (%) was monitored with CellTiter-Glo luminance^48^.

### Anti-inflammatory activities and inhibitory effects against MMP-1 and MMP-9 on UV-induced cellular damaged in the coculture model

Interleukins (IL), i.e., IL-6 and IL-8 secretions in the coculture system were examined at the noncytotoxic concentration of the sample in a comparison with the control groups, i.e., vehicle control and non-UV exposure. In addition, the secreted MMP-1 and MMP-9 were examined in parallel^48^.

### Safety assessment in human ex vivo skin model

Primary HDF were obtained from abdominal biopsies (Plastic Surgery, Yanhee International Hospital, Thailand), with the written informed consent of donors and approval by the hospital ethical committee according to the principles of the Declaration of Helsinki. Aged ex vivo skin tissues were obtained from the abdominal region from a person who had undertaken cosmetic surgery. The tissues were collected during a surgical procedure from a healthy Thai female donor of more than 35 years old. The tissues were maintained in a culture medium containing DMEM and supplements. The samples were daily applied onto the tissues for a consecutively 7 days. The cell viability of the skin tissues was examined at 570 nm^49^.

### Activities on hyaluronic acid, tropocollagen, IL-6 and MMP-1 in human ex vivo skin model

The quantity of hyaluronic acid, tropocollagen, IL-6 and MMP-1 in the human ex vivo skin model was examined by the method of Klinngam and coworkers^50^. Briefly, the tissue culture medium was harvested following a consecutive 7-day of daily treatment with the sample at the safe dose. The culture medium was harvested, centrifuged and analyzed by ELISA using hyaluronic acid (AL254HVc; PerkinElmer, MA, USA), procollagen type I C-peptide (PIP, AL353HVc; PerkinElmer), IL-6 (AL223c; PerkinElmer) and MMP-1 (AL242c; PerkinElmer) kits. Triplicate assessments were undertaken with 2 tissues per treatment.

### Stability assessment

The extract was challenged on its stability following storages at different temperatures, i.e., 4 °C, room temperature (RT) and 45 °C for 6 months^12,13^. Which, color (CIELAB system; Colorimeter UltraScanVIS, USA) of the extract was monitored every month in addition to HPLC analysis of catechins.

### Statistical analysis

Data are presented as the mean ± SD. The results from the cellular study models are expressed as the mean ± SEM. The parameters were compared and analyzed using ANOVA test with a significance level of p < 0.05 using the SPSS program version 16.0.

## Data Availability

The data used to support the findings of this study are available from the corresponding author upon a reasonable request.

## Abbreviations

ABTS: 2,2′–Azino-bis(3-ethylbenzothaiazoline)-6-sulfonic acid)
AcCN: Acetonitrile
ATE: Assam tea extract
DPPH: 1,1-Diphenyl-2-picrylhydrazyl
C: Catechin
CF: Caffeine
CG: Catechin gallate
EC: Epicatechin
ECG: Epicatechin-3-gallate
ECM: Extracellular matrix
EGC: Epigallocatechin
EGCG: Epigallocatechin-3-gallate
EqC: Equivalent concentration
EtOH: Ethanol
FBS: Fetal bovine serum
FRAP: Ferric reducing ability of plasma
GC: Gallocatechin
GCG: Gallocatechin gallate
HDF: Human dermal fibroblast
HPLC: High performance liquid chromatography
IC_50_: Inhibitory concentration at 50%
IL: Interleukin
Int.: Initial
MeOH: Methanol
MMP: Matrix metalloproteinase
PM: Particulate matter
RT: Room temperature
SRB: Sulforhodamine B
TFA: Trifluoroacetic acid
TFC: Total flavonoid content
TPC: Total phenolic content
TPTZ: 2,4,6-Tri(2-pyridyl)-*S*-triazine
TRP: Tyrosinase related protein
TTC: Total tannin content
